# Supplementary material for: Ozone exposure disrupts insect sexual communication
Source: Nat Commun. 2023 Mar 14;14:1186. doi: 10.1038/s41467-023-36534-9 (PMC10014992; doi:10.1038/s41467-023-36534-9)
Supplement: Supplementary file 1 — Supplementary Information [file 41467_2023_36534_MOESM1_ESM.pdf]

Supplementary Materials for

Title: Ozone exposure disrupts insect sexual communication

Authors: Nan-Ji Jiang[1,2], Hetan Chang[1], Jerrit Weißflog[3], Franziska Eberl[4], Daniel Veit[4], Kerstin Weniger[1], Bill S. Hansson[1,2#], Markus Knaden[1,2#*]

*Corresponding author.
E-mail: mknaden @ice.mpg.de

This PDF file includes:

Supplementary Figures and Figure legends
Captions for Movies S1 and S2

Other Supplementary Materials for this manuscript include the following:

Movies S1 and S2

Supplementary Figures and Figure Legends

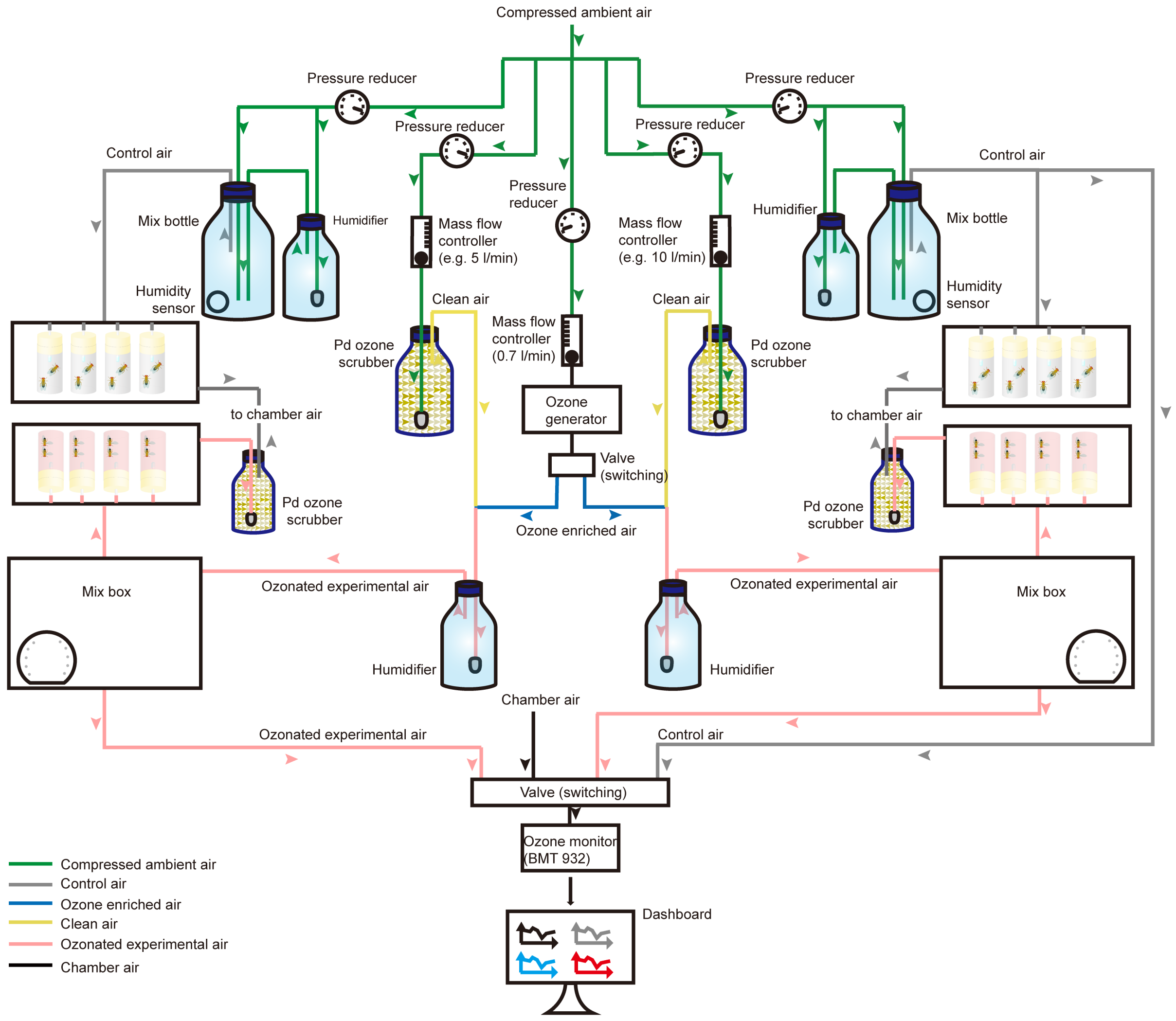

Fig. S1. The ozone exposure system. Schematic of the device to produce defined levels of ozone. For a detailed description of its function, please see the methods part of the main manuscript.

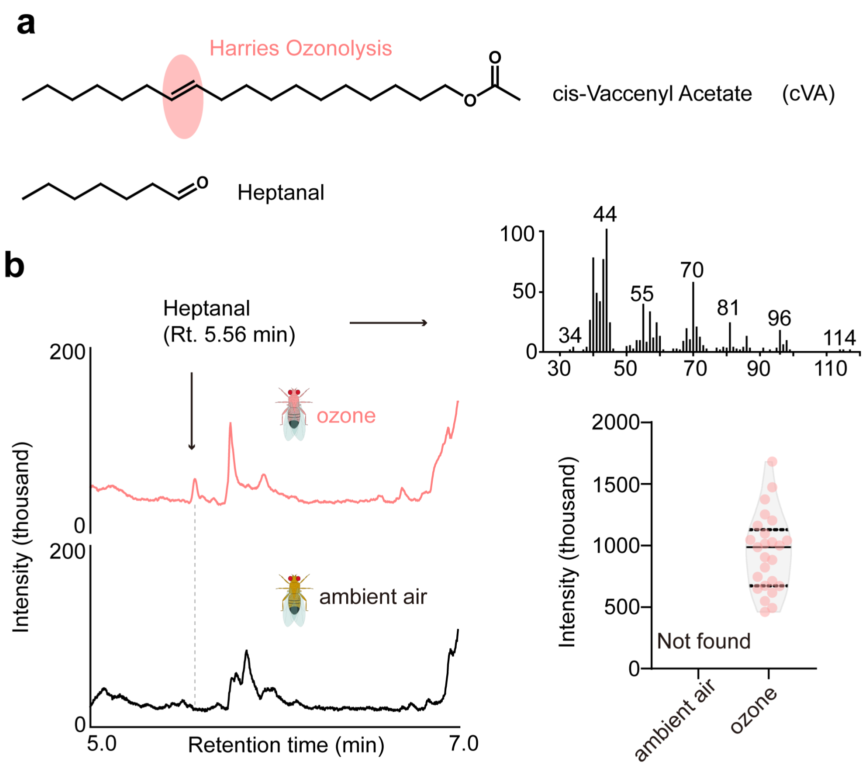

Fig. S2. Ozone-exposed *D. melanogaster* (CS) males emit more heptanal. a, Potential degradation of cVA following Harries Ozonolysis. **b**, Chromatograms (left) and MS spectrum (right up) and quantification (right down) of heptanal from males exposed to ozone or ambient air. N=25 for air exposure or 100ppb ozone exposure.

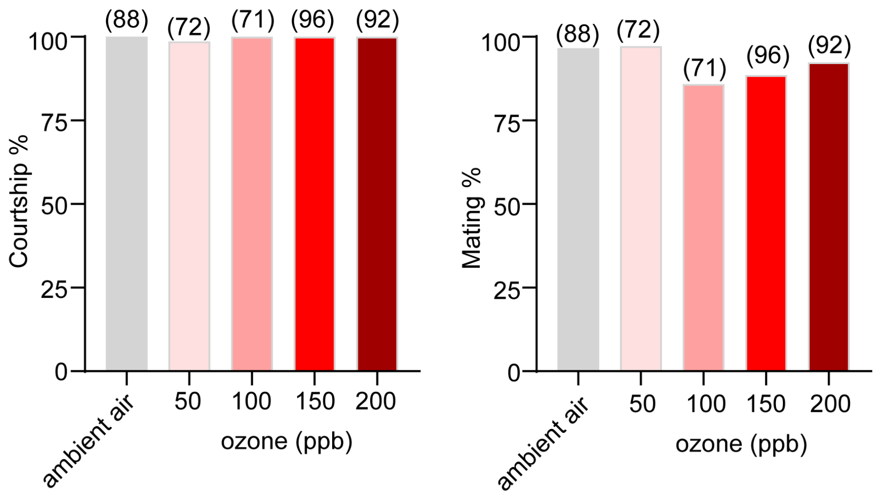

Fig. S3. Effect of *D. melanogaster* (CS) male exposure for 30 min to different levels ozone on courtship behavior. Courtship percentage (i.e. percentage of experiments resulting in courtship behavior) and mating success (i.e. percentage of experiments resulting in mating). Sample sizes provided in brackets, no significant differences from control ambient air control group (Two-tailed *Fisher's exact* test with *Holm-Bonferroni* correction for multiple comparison with control group).

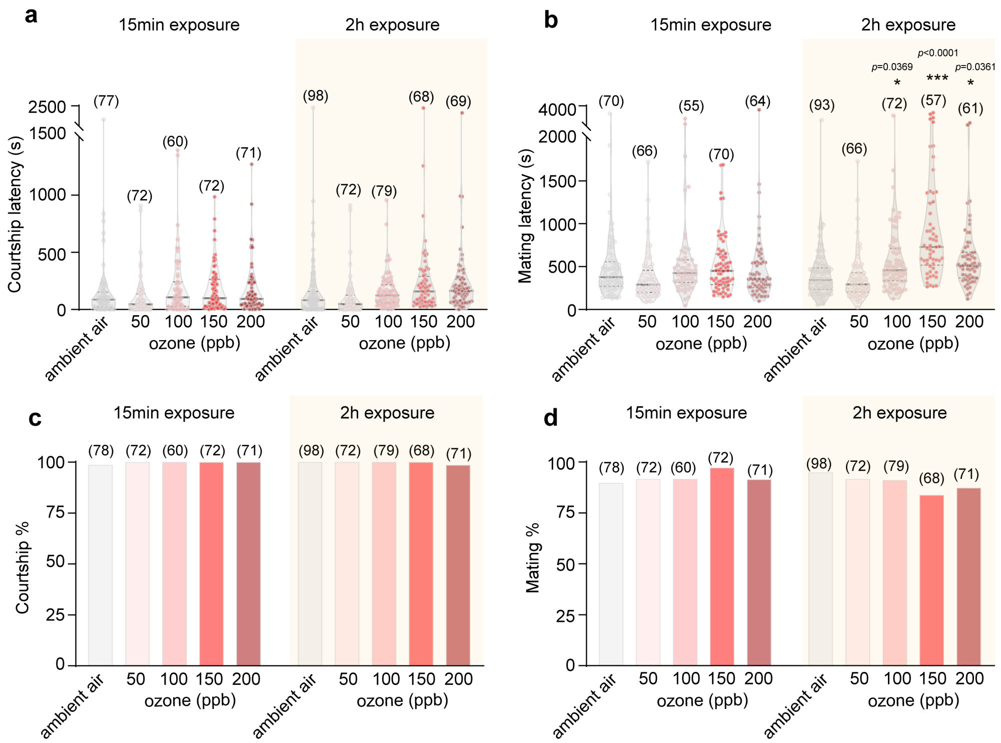

Fig. S4. Effect of different exposure times and levels of ozone on courtship behavior of *D. melanogaster* (CS) males. a, Courtship latency (i.e. time until male starts courting). **b**, Mating latency (i.e. time until mating starts). **c**, Courtship percentage (i.e. percentage of experiments resulting in courtship behavior). **d**, Mating success (i.e. percentage of experiments resulting in mating). Samples are given in brackets. (**a** and **c**, *Dunnett's* test for multiple comparisons against ambient air control group; **b** and **d**, *Fisher's exact* test with *Holm-Bonferroni* correction for multiple comparison with control group, *p<0.05; **p<0.01; ***p<0.001). All tests are two-tailed.

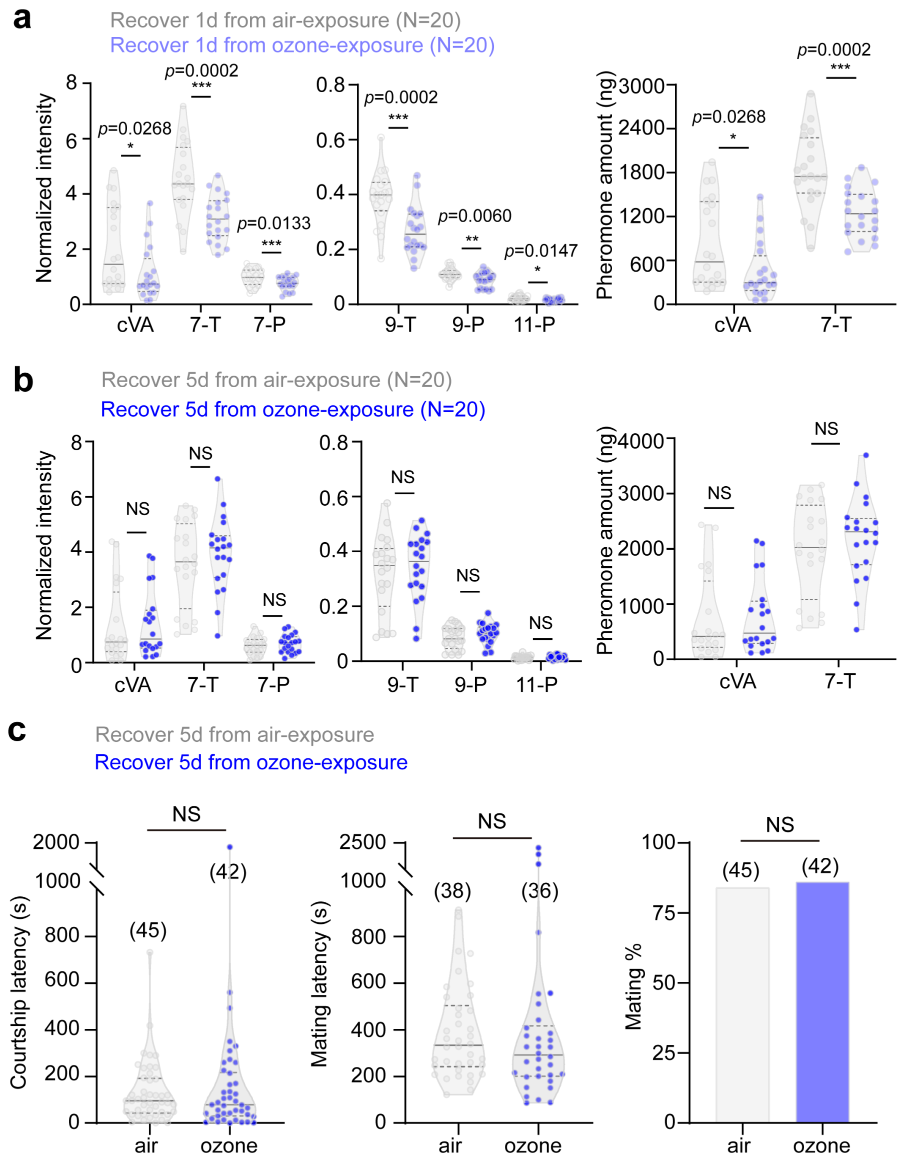

Fig. S5. Recovery of chemical profiles after *D. melanogaster* (CS) males have been exposed 2h to 100ppb ozone. a, One day after exposure, male compounds of ozone-exposed flies still differ from control males. **b**, Five days after exposure, chemical profiles are fully recovered. cVA: cis-vaccenyl acetate; 7-T: (*Z*)-7-Tricosene; 9-T: (*Z*)-9-Tricosene; 7-P: (*Z*)-7-Pentacosene; 9-P: (*Z*)-9-Pentacosene; 11-P: (*Z*)-11-Pentacosene. Data depict normalized intensity for the different described pheromones of *D. melanogaster* males and the quantified amount of cVA and 7-T. **c**, Five days after exposure courtship behavior is fully recovered. (**a-c**) Two-tailed unpaired *t*-test; NS indicates no significant difference, *$p<0.05$; **$p<0.01$; ***$p<0.001$.

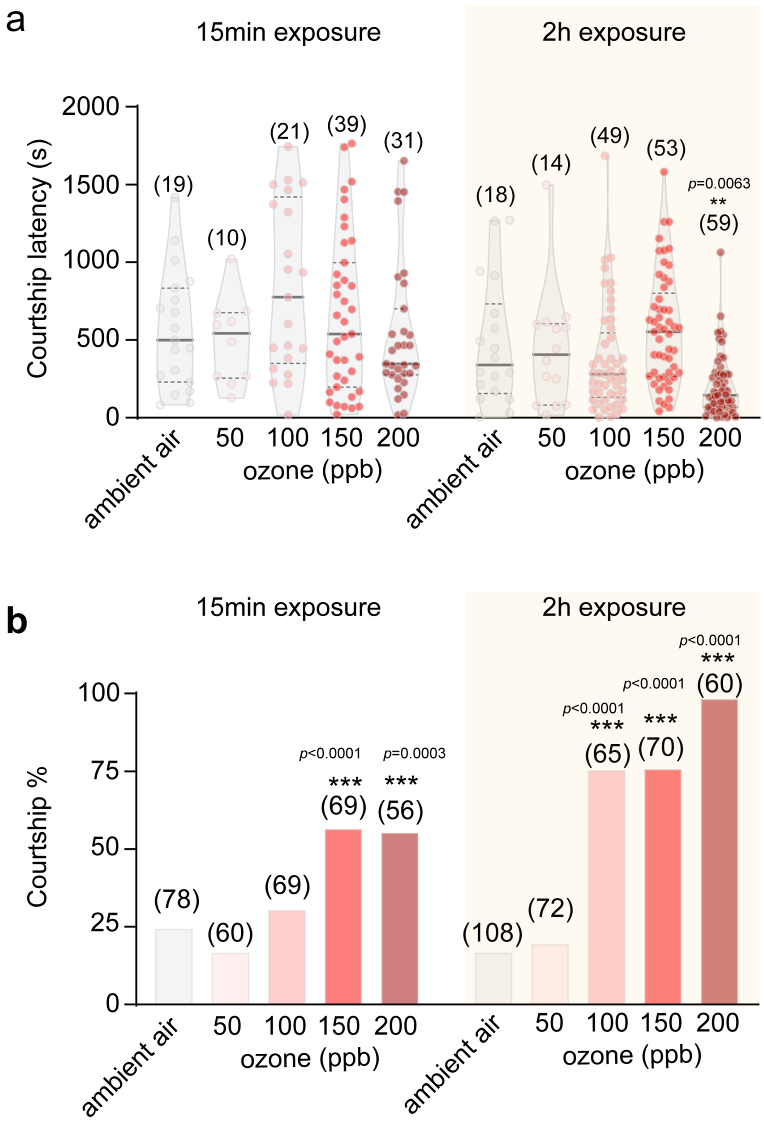

Fig. S6. Ozone induces *D. melanogaster* (CS) male-male courtship behavior. a, Courtship latency (i.e. time until courtship behavior starts) after 15min (left panel) or 2h (right panel) exposure to different levels of ozone. Sample sizes are provided in brackets. Two-tailed *Dunnett's* test for multiple comparisons against the ambient air control; group significantly differing from control: $^{**}p<0.01$. **b**, Courtship percentage (i.e. percentage of experiments that result in courtship within 10min). Two-tailed *Fisher's exact* test with *Holm-Bonferroni* correction for multiple comparison with control group, $^{***}p<0.001$.

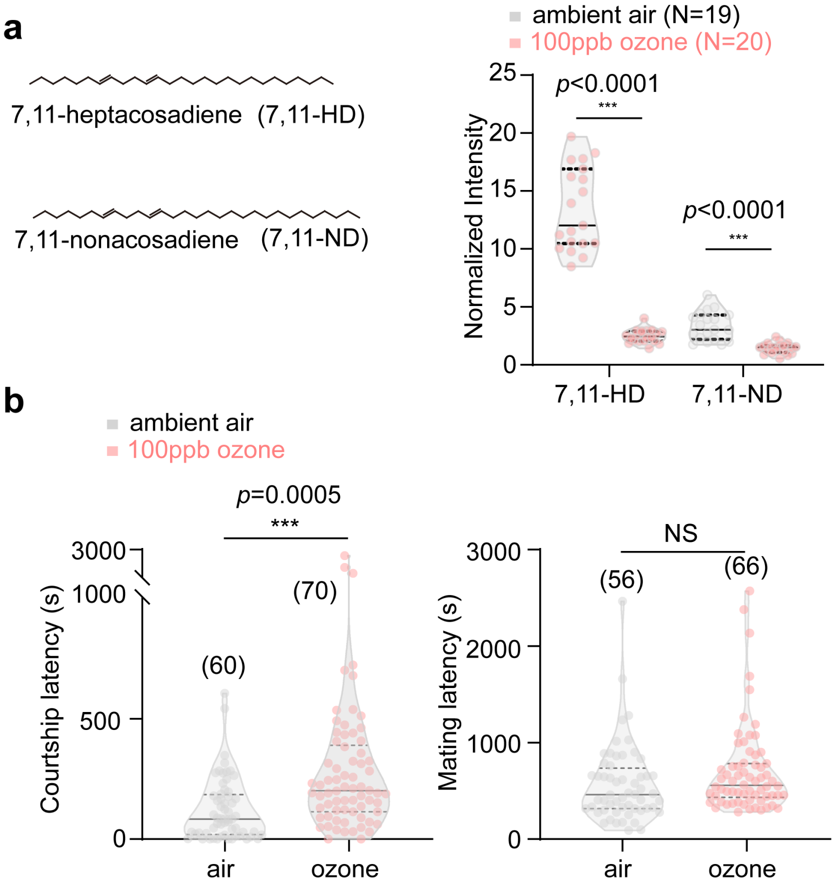

Fig. S7. Exposure of *D. melanogaster* (CS) female flies to ozone changes their chemical profiles and attractiveness to males. a, Normalized intensity of 7,11-Heptacosadiene (7,11-HD) and 7,11-Nonacosadiene (7,11-ND) in ozone exposed and control flies (Two-tailed unpaired *t*-test; ****p*<0.001). **b**, Courtship and mating latency of males confronted with ozone-exposed and control females (Two-tailed unpaired *t*-test; NS indicates no significant difference; ****p*<0.001).

Supplementary Movie 1.

Courtship-chain formation of *D. melanogaster* males during ozone exposure. Males were exposed to 100 ppb ozone for ca. 20 min.

Supplementary Movie 2.

No male-male courtship of *D. melanogaster* males during exposure to control air.
