# Supplementary material for: Ozone exposure disrupts insect sexual communication
Source: Nat Commun. 2023 Mar 14;14:1186. doi: 10.1038/s41467-023-36534-9 (PMC10014992; doi:10.1038/s41467-023-36534-9)
Supplement: Supplementary file 2 — Reporting Summary [file 41467_2023_36534_MOESM2_ESM.pdf]

nature portfolio

Corresponding author(s): Markus Knaden

Last updated by author(s): Jan 18, 2023

Reporting Summary

Nature Portfolio wishes to improve the reproducibility of the work that we publish. This form provides structure for consistency and transparency in reporting. For further information on Nature Portfolio policies, see our Editorial Policies and the Editorial Policy Checklist.

Statistics

For all statistical analyses, confirm that the following items are present in the figure legend, table legend, main text, or Methods section.

n/a	Confirmed	
[ ]	[x]	The exact sample size (*n*) for each experimental group/condition, given as a discrete number and unit of measurement
[ ]	[x]	A statement on whether measurements were taken from distinct samples or whether the same sample was measured repeatedly
[ ]	[x]	The statistical test(s) used AND whether they are one- or two-sided<br>*Only common tests should be described solely by name; describe more complex techniques in the Methods section.*
[ ]	[x]	A description of all covariates tested
[ ]	[x]	A description of any assumptions or corrections, such as tests of normality and adjustment for multiple comparisons
[ ]	[x]	A full description of the statistical parameters including central tendency (e.g. means) or other basic estimates (e.g. regression coefficient) AND variation (e.g. standard deviation) or associated estimates of uncertainty (e.g. confidence intervals)
[ ]	[x]	For null hypothesis testing, the test statistic (e.g. *F*, *t*, *r*) with confidence intervals, effect sizes, degrees of freedom and *P* value noted<br>*Give P values as exact values whenever suitable.*
[x]	[ ]	For Bayesian analysis, information on the choice of priors and Markov chain Monte Carlo settings
[x]	[ ]	For hierarchical and complex designs, identification of the appropriate level for tests and full reporting of outcomes
[x]	[ ]	Estimates of effect sizes (e.g. Cohen's *d*, Pearson's *r*), indicating how they were calculated

Our web collection on statistics for biologists contains articles on many of the points above.

Software and code

Policy information about availability of computer code

Data collection	Behavioral data were collected by visual observation. Chemical data were collected by GC-MS
Data analysis	Statistical analysis was performed with Graph Pad Prism Version 8. Chemical analysis was performed with MSD ChemStation E. 02.01.1117

For manuscripts utilizing custom algorithms or software that are central to the research but not yet described in published literature, software must be made available to editors and reviewers. We strongly encourage code deposition in a community repository (e.g. GitHub). See the Nature Portfolio guidelines for submitting code & software for further information.

Data

Policy information about availability of data

All manuscripts must include a data availability statement. This statement should provide the following information, where applicable:

- Accession codes, unique identifiers, or web links for publicly available datasets
- A description of any restrictions on data availability
- For clinical datasets or third party data, please ensure that the statement adheres to our policy

All data is available in the main text or the supplemental material. Data statement is added to the manuscript.

Human research participants

Policy information about studies involving human research participants and Sex and Gender in Research.

Reporting on sex and gender	n/a
Population characteristics	n/a
Recruitment	n/a
Ethics oversight	n/a

Note that full information on the approval of the study protocol must also be provided in the manuscript.

Field-specific reporting

Please select the one below that is the best fit for your research. If you are not sure, read the appropriate sections before making your selection.

[x] Life sciences   [ ] Behavioural & social sciences   [ ] Ecological, evolutionary & environmental sciences

For a reference copy of the document with all sections, see nature.com/documents/nr-reporting-summary-flat.pdf

Life sciences study design

All studies must disclose on these points even when the disclosure is negative.

Sample size	Sample sizes were chosen to at least match or rather exceed those of similar publications (e.g. Dweck et al. PNAS 2015 Pheromones mediating copulation and attraction in Drosophila)
Data exclusions	no data was excluded from the analysis
Replication	All numbers of replications are provided as sample sizes in the different figures.
Randomization	Animals that were either exposed or not exposed to ozone were always collected from the same vials (i.e. population of flies)
Blinding	Whenever possible, behavioral assays were performed without knowing, to which treatment group the animals belonged to. All chemical analyses (i.e. pheromone quantifications) of flies were performed without knowing, to which treatment group an animal belonged to.

Reporting for specific materials, systems and methods

We require information from authors about some types of materials, experimental systems and methods used in many studies. Here, indicate whether each material, system or method listed is relevant to your study. If you are not sure if a list item applies to your research, read the appropriate section before selecting a response.

Materials & experimental systems

n/a	Involved in the study	
[x]	[ ]	Antibodies
[x]	[ ]	Eukaryotic cell lines
[x]	[ ]	Palaeontology and archaeology
[ ]	[x]	Animals and other organisms
[x]	[ ]	Clinical data
[x]	[ ]	Dual use research of concern

Methods

n/a	Involved in the study	
[x]	[ ]	ChIP-seq
[x]	[ ]	Flow cytometry
[x]	[ ]	MRI-based neuroimaging

Animals and other research organisms

Policy information about studies involving animals; ARRIVE guidelines recommended for reporting animal research, and Sex and Gender in Research

Laboratory animals	Drosophila putrida (15081-1401.00 / 15100-1711.01), D. repletoides (E-17001), D. funebris (15120-1911.05), D. pallidipennis (15210-2331.02), D. saltans (14028-0571.00 / 15250-2451.01), D. moj. wrigleyi (15081-1352.22), D. moj. mojavensis (15081-1352.47), D. busckii (1300-0081.00), D. suzukii (14029-0011.01 / 14011-0131.04). For experiments with D. melanogaster we

	used the wild type strain Canton-S (CS). All tested animals were 10 days old.
Wild animals	no wild animals were tested
Reporting on sex	The main experiments were performed with male flies as females decide for a copulation based on male pheromones. However we also analyzed the effect of ozone on female flies.
Field-collected samples	no samples were collected from the field
Ethics oversight	n/a as all experiments were performed with flies

Note that full information on the approval of the study protocol must also be provided in the manuscript.
