# Supplementary material for: Ozone exposure disrupts insect sexual communication
Source: Nat Commun. 2023 Mar 14;14:1186. doi: 10.1038/s41467-023-36534-9 (PMC10014992; doi:10.1038/s41467-023-36534-9)
Supplement: Supplementary file 3 — Description of Additional Supplementary Files [file 41467_2023_36534_MOESM3_ESM.docx]

**Description of Additional Supplementary Files**

Supplementary Movie 1

Description: Courtship-chain formation of *D. melanogaster* males during ozone exposure. Males were exposed to 100 ppb ozone for ca. 20 min.

Supplementary Movie 2

Description: No male-male courtship of D. melanogaster males during exposure to control air.
